# Stigmatizing attitudes towards depression among university students in Syria

**DOI:** 10.1371/journal.pone.0273483

**Published:** 2022-09-15

**Authors:** Sarya Swed, Sheikh Sohib, Noheir Ashraf Ibrahem Fathy Hassan, Mohammad Badr Almoshantaf, Sidra Mhd Sammer Alkadi, Yossef Hassan AbdelQadir, Nancy Ibrahim, Lina Taha Khair, Agyad Bakkour, Ali Hadi Hussein Muwaili, Dhuha Hadi Hussein Muwaili, Fatima Abubaker Abdalla Abdelmajid, Eman Mohammed Sharif Ahmad, Muhammad Mainuddin Patwary, Bisher Sawaf, Mhd Kutaiba Albuni, Elias Battikh, Nashaat Kamal Hamdy Elkalagi

**Affiliations:** 1 Faculty of Medicine, Aleppo University, Aleppo, Syria; 2 Department of Psychiatry, Jawahar Lal Nehru Memorial Hospital, Srinagar, Kashmir, India; 3 Faculty of Medicine, Aswan University, Aswan, Egypt; 4 Department of Neurosurgery, Ibn Al-Nafess Hospital, Damascus, Syria; 5 Faculty of Medicine, Damascus University, Damascus, Syria; 6 Faculty of Medicine, Alexandria University, Alexandria, Egypt; 7 The National Ribat University, Al-Ribat, Sudan; 8 Faculty of Medicine, Albaath University, Homs, Syria; 9 Ivano-Frankivsk National Medical University, Ivano-Frankivsk, Ukrain; 10 University of Medical Sciences and Technology, Khartoum, Sudan; 11 Nile Valley University, Atbra, Sudan; 12 Environment and Sustainability Research Initiative, Khulna, Bangladesh; 13 Environmental Science Discipline, Life Science School, Kulna University, Khulna, Bangladesh; 14 Department of Internal Medicine, Syrian Private University, Damascus, Syria; 15 Internal Medicine and Tropical Medicine at Faculty of Medicine, Al Arish University, Al Shaba, Egypt; George Mason University, UNITED STATES

## Abstract

**Background:**

Depression is a prominent cause of mental disability globally, having a severe impact on mental and physical health. Depression rehabilitation and treatment, whether through psychiatric management or counseling therapy, is hampered by stigmatizing attitudes regarding psychiatric illness patients impacted by societal and cultural factors. However, little is known about the stigma toward people with depression among the students in Syria.

**Methodology:**

A total of 1,056 students in Syria completed a questionnaire that included a case narrative illustrating depression. A total of 1,056 students in Syria completed a questionnaire that included a case narrative illustrating depression. The survey looked at attitudes toward depression, the desire to keep a safe distance from depressed people, stigma attitudes toward people with depression among college students, perceived beliefs about depressive people, gender (male and female), and the major section (medical and medical and non-medical) differences.

**Results:**

Four questionnaires have refused to finish the survey, out of 1259 issued. Around 47.80% of respondents, most of whom were females, felt that sad people might snap out of it. 14.60 percent believe depression isn’t even an actual medical condition. Surprisingly, 2% of respondents with a medical background thought the same thing. Regarding more extreme stigmatization, 16.80% of respondents thought depressed persons were harmful. People with depression will be avoided by 19.50 percent of respondents, and people with medical backgrounds will be avoided by 5.20 percent of respondents. Nearly one-fifth of those polled said they would not tell anyone if they were depressed. Only a tiny percentage of respondents (6.90 percent) said they would not hire or vote for a politician who suffers from depression (8.40 percent).

**Conclusion:**

According to the study, Syrian college students had a significant level of stigma and social distance toward mentally ill patients. Female students and non-medical students had a higher stigma in most subscale items for people with depression.

## Introduction

Most people define "Depression" as a feeling of loss or being sad; however, it is more than that. Depression is a severe psychiatric condition that is highly prevalent worldwide [1]. WHO has ranked Major Depressive Disorders (M.D.D.) as the third cause of the burden of disease worldwide in 2008, and it is expected that it will be ranked first by 2030 [2]. About 350 million people of all ages worldwide are affected by depression [3]. The prevalence of Depression has risen recently, especially in children and adolescents, as shown by the U.S. national comorbidity survey, which found that between 2001 to 2004, about 11.7% of adolescents aged 13 to 18 years have met the criteria for major depressive disorder. Also, the 12-month prevalence of major depressive episodes has increased from 8.7% in 2005 to 11.3% in 2014 in adolescents and from 8.8% to 9.6% in young adults [4]. Researchers have also shown higher rates of Depression among university students compared to the general population [5, 6]; a Chinese study shows that about 23.8% of university students are affected by depression [7].

Although the exact cause of Depression is still unknown, studies show that the etiology of Depression is multifactorial, including Biological and environmental factors and psychological factors. Biological factors include genetic elements as the heritability of Depression is estimated to be approximately 35%, and hormones or abnormalities in neurotransmitters such as dopamine and norepinephrine are also involved in the etiology of M.D.D. Environmental factors include environmental stressors, sexual, physical, or emotional abuse during childhood, associated with the high risk of developing M.D.D. [2, 8]. Diagnosis of M.D.D. It depends on D.S.M.’s 5 diagnostic criteria, which require the presence of 5 of 9 symptoms for 2 weeks, provided that one of these symptoms must be the depressed mode or anhedonia [9]. Depression is associated with a high mortality rate and shorter life expectancy [10, 11]; an increased risk of suicide can explain this among affected people [12, 13], and a bad prognosis of other medical conditions lead to an increased risk of developing complication and high risk of Mortality [14]. Depression has chronic nature and requires lifelong treatment to diminish relapse and recurrence rates [15]. However, people affected by Depression suffer from stigmatizing beliefs [16] which may be considered a barrier to recovery.

Stigma means that people would distinguish one negatively, a pejorative attribute implying shame. When related to mental illness, it generates fear of being excluded or rejected by others due to false beliefs arising from a lack of knowledge about the nature of mental illness [17, 18]. Mentally ill people such as those affected by depression fight in two ways first, they fight against their disease and its symptoms affecting their lives; second, they fight against the general public stigmatizing attitudes against them, pushing them to be set apart from the others, causing them to feel lonely and aggravate their condition. Stigma is classified into public and self-stigma; public stigma is the attitude of the general population toward people with mental illness. In contrast, self-stigma is the internalized sensation of shame that those people have about themselves [19]. It has been reported that stigmatization of mental illness, including depressive disorders, is still highly prevalent [20]. Most mentally ill people express negative attitudes against them from others, such as (avoidance, forcing them for treatment, setting them isolated from the others, hostile behavior). This is almost because the general public considers them dangerous and responsible for their behavior (controllability and responsibility) [21]. Harmful consequences of stigmatized attitudes have been reported by WHO, including destroyed family relationships and social exclusion [22] As stigma against mental illness is highly prevalent [23], depressed people, like other mentally ill patients, suffer from stigmatizing and discriminative attitudes against them, making them embarrassed about seeking help leading to more deterioration in their condition which may affect their daily life, they prefer not to ask for help as they think that the others will distinguish them negatively if they sought such help [24, 25]. A Canadian study reported that people with mental illness using health services suffer from stigmatizing attitudes [26]. A study held in the School of Medicine at the University of California, San Francisco, showed that only 22% of students suffering from Depression were ready to receive counseling services, while others were not [27]. Studies show a high prevalence of perceived stigma of Depression among students [28], as demonstrated by Chinese research that reported high levels of stigmatizing attitudes towards Depression are prevalent among the Chinese public, with about 53.0% of participants reporting personal stigma and 83.4% reported perceived stigma [29], In addition, medical students show a high prevalence of stigma against depression [30]. To face stigma and discrimination, Anti-stigma initiatives have been set up to reduce their harmful effects on patients and improve the social understanding of mental illness [31].

By checking the literature, we found no similar studies in Syria that assess the stigma among the population toward mental health patients. However, some studies have followed post-traumatic self-stigmitizing Syrians in asylum countries [32, 33]. Similar studies have taken part in Arab countries like Saudi, Lebanon, and Oman [34–36]. Also, there is a considerable gap related to suitable and supported management of mental illnesses patients by citizens in Arab countries, especially low-income countries such as Syria.

Our goal was to establish the frequency of public stigma against Depression in a sample of Syrian students, including personal and perceived stigma and the explanations behind these stigmata. We hypothesized that, given the high prevalence of stigma attitudes toward Depression, stigma attitudes toward Depression are prevalent among Syrian students, so we conducted this study to determine: 1) whether stigma is prevalent among Syrian students and 2) the degree of stigmatizing attitudes and social distancing among them. The fighting stigma connected with Depression is a vital method of enhancing mental and physical health and offering those suffering in silence a voice. To make all communities and community members safer and healthier, stigma must be eliminated. So we recommend paying attention to the disastrous effect of stigma on depressed individuals and the importance of developing anti-stigma programs in health facilities with more future investment in health facility stigma reduction.

## Methods

### Study design

We conducted an online cross-sectional to collect data from Syrian university students using an online questionnaire based on similar research [37] in the literature. We modified the survey and translated it into the Arabic language in a suitable form for Syrian students. The used questionnaire was uploaded in S1 Table.

### Setting and participants

1259 Syrian students from different universities took part in this survey and filled out a Google Form website questionnaire. The data collection duration lasted from 18th to 27th March 2021. Only the Syrian students above 18 years were eligible to participate in the study.

To acquire the requisite responders, convenience and snowball strategies were used. The online questionnaire was shared on social media groups such as WhatsApp, Facebook, and telegram by collaborators to gather the responses. We didn’t perform a statistical power analysis to calculate the required sample size because there are no accurate reports of the number of Syrian students. Twenty-five random responses were gathered to study if the questionnaire was suitable and easy to understand and answer or not, and we conducted some modifications depending on the participants’ comments.

We also conducted a pilot analysis involving 40 responses to check that the questionnaire’s reliability was validated. The Cronbach’s alpha values range from 0.712 to 0.861, demonstrating that the used tool had a high internal consistency level. We published the questionnaire once the pilot study was finished.

### Measurements in the survey questionnaires

The questionnaires consisted of seven parts: the first part was a range of questions about demographic data like age, gender and social status, the second part was questions about personal stigma toward depression scale, the third part includes questions about perceived stigma towards depression scale, the fourth part was closed-ended questions about social distance with depressed people with Yes or No answers, the fifth part consists of questions about the participants’ usual source of their knowledge about depression like newspapers, TV or websites, the sixth part is concerned by helpfulness or intervention, this part is subdivided into four subgroups of question with multiple answer, these subgroups are People who can help, Medications which can help, other interventions and help methods, the last part is concerned by supporting information, it includes three cases participants should answer in order to give confirmation about their knowledge towards 3 mental health disorders (Depression, schizophrenia and anxiety). The used questionniare was uploaded as a suplemanteray material.

### Depression Stigma Scales

The DSS contains personal stigma subscales (nine items) and perceived stigma subscales (nine items). The statements in each item of the two subscales are the same except for the subject of items. In the personal stigma subscales, respondents were asked about their attitude toward people with depression symptoms described in the vignette (e.g., “People with depression could snap out of it if they wanted”). In the perceived stigma subscales, respondents were asked their beliefs about most of the other people’s attitudes toward people with depression symptoms described in the vignette (e.g., “Most people believe that people with depression could snap out of it if they wanted”). The response of each item was measured on a five-point scale ranging from “strongly agree” to “strongly disagree” [32]. The Chinese version of the scale has been widely used with good reliability and validity [38].

There are two sections in the questionnaire that explain the Participants’ usual sources of mental health knowledge, especially depression, and the recommended opinions towards supporting interventions for helping depressed persons.

In addition, this questionnaire includes a vignette of 3 patients with mental illnesses (depression, schizophrenia, and GAD) to assess the students’ knowledge of the diagnosis of these psychiatric disorders.

### Social distance scale

The five-item short measurement of SDS was developed by [39] to measure the desire for social distancing from a person with mental illness. The Chinese version of the SDS was used to estimate the willingness to come into contact (such as live next door, marry into the family) with the person described in the vignette. The response of each item was measured on a four-point scale, which ranged from “definitely willing” to “definitely unwilling.” The reliability and validity of its Chinese version have been tested, and the results showed that all the indicators met the requirements of psychometrics.

The used questionnaire was uploaded as suplementrail file.

### Ethics statement

The protocol was approved by the dean of Damascus University and Aleppo University in March 2021. The convenience sampling method was used in the present study. Considering the sample’s representativeness, this study randomly selected different classes by the school, grade, and significance. The aim of the present study was explained in the questionnaires, and oral informed consent was obtained from all the respondents. They were encouraged to independently analyze the vignette and answer a battery of questions, including demographic information, depression stigma scales (DSS), and social distance scale SDS. The survey contained a cover page stating responses were anonymous and voluntary and would have no impact on the participants.

### Statistical analysis

All data were analyzed by SPSS 28 and Excel. Descriptive statistics, including demographic characteristics, Participants’ usual sources of mental health knowledge, and helpful interventions, were presented as numbers and percentages. Still, continuous variables were presented as mean and standard deviation. Stigma attitudes toward people with depression (percentage frequencies and 95% CI) and social distance (percentage frequencies and 95% CI). The options of “agree” and “strongly agree” were combined into one option on the DSS, and the possibilities of “Yes” and “No” were combined into one option on the SDS. The integrated options represent the positive and negative attitudes of the respondents. Sperman and one-way ANOVA test were used to assess the significant difference in each item on the three scales that assess stigma towards depression patients and social distance scale among different demographic variables (gender, major, educational level, and school level) in the proportion of agreement. Multiple logistic regression was conducted to study the predicted relationship between the three scales and other demographic variables. We considered the response with half of the total score or under equal 0 value, but the answer with above half of the total score equals 1vaule. The value of p was set at <0.05 for statistical significance.

## Results

### Demographic baseline characteristics of the study sample

Out of 1259 distributed questionnaires, four questionnaires have refused to complete the survey. The baseline characteristics of the participants are shown in Table 1. The average age of the respondents was 22.4± 3.85 (mean ± SD). The ratio of gender (male 26%: female 74%) was approximately 1:3, which may be attributed to the already imbalanced gender ratio in Syrian schools. To explore the difference in stigmatizing attitudes between medical and non-medical students, we have classified students’ majors into medical majors (34.8%) and non-medical majors (65.2%). About 43.1% of students were working during their educational lives. More than half of respondents (58.2%) have reported a positive history of mental health disease, but only (7%) are under psychological treatment.

**Table 1:** Baseline characteristics of the participants.

	N = 1259	
	n	%
**Age (Mean/SD)**	**22.4/3.85**	
**Gender**		
**Male**	327	26.0%
**Female**	932	74.0%
**Social status**		
**Single**	1095	87.0%
**Married**	125	9.9%
**Divorced**	23	1.8%
**Widower**	16	1.3%
**Major section**		
**Medical student**	438	34.8%
**Non-medical student**	821	65.2%
**Economic level**		
**Bad**	105	8.3%
**Middle**	748	59.4%
**Good**	363	28.8%
**High**	43	3.4%
**The University stage**		
**1^ST^ year**	229	18.2%
**2^nd^ year**	216	17.2%
**3^rd^ year**	204	16.2%
**4^th^ year**	262	20.8%
**5^th^ year**	186	14.8%
**6^th^ year**	162	12.9%
**Region**		
**City**	890	70.7%
**Rural**	369	29.3%
**Occupation status**		
**Worker**	542	43.1%
**Non-Worker**	717	56.9%
**Live with**		
**Family**	1051	83.5%
**With father**	27	2.1%
**With mother**	103	8.2%
**With friends**	78	6.2%
**Immigrant status**		
**Yes**	560	44.5%
**No**	699	55.5%
**History of Mental Health Disease**		
**Yes**	733	58.2%
**No**	526	41.8%
**Current psychological treatment**		
**Yes**	88	7.0%
**No**	1171	93.0%
**Current pharmacological treatment**		
**Yes**	174	7.0%
**No**	1085	93.0%

### Personal stigma

In Table 2, we collected the gender differences and medical and non-medical significant differences in the percentage of participants who held stigma attitudes toward the person with depression. About 47.80% of respondents agreed that depressed people could snap out of the problem; most were females. 14.60% stated that depression is not even an actual medical illness. Surprisingly enough, 2% of medical background respondents thought that as well. As for more extreme stigmatization, 16.80% of respondents considered depressed people as dangerous people. 19.50% will tend to avoid people with depression, and 5.20% of medical background respondents will also prevent these people. Approximately 18.80% of respondents won’t tell anyone if they suffer from depression. Only a small portion of respondents won’t hire anyone with depression (6.90%) or elect a politician suffering from depression (8.40).

**Table 2:** Percentage of participants who “agree” or “strongly agree” with about personal stigma towards depression patient scale statements.

**Statement about personal belief**	**Total (N = 935)**		**Gender**				**Major section**				**Region**				**Economic level**								**Occupation status**			
	**n**		**Male (n = 327)**		**Female (n = 932)**		**Medical (n = 438)**		**Non-Medical (n = 821)**		**City (n = 890)**		**Rural region (n = 369)**		**Low (n = 105)**		**Moderate (n = 748)**		**Good (n = 363)**		**High (n = 43)**		**Worker (n = 542)**		**Non-worker (n = 717)**	
		**%**	**n**	**%**	**n**	**%**	**n**	**%**	**n**	**%**	**n**	**%**	**n**	**%**	**n**	**%**	**n**	**%**	**n**	**%**	**n**	**%**	**n**	**%**	**n**	**%**
**The person could snap out of the problem**	**602**	**47.80%**	**115**	**9.20%**	**477**	38.2%**	**179**	**14.30%**	**413**	33.1%**	**421**	**33.70%**	**171**	**13.70%**	**37**	**3.00%**	**370**	**29.60%**	**171**	**13.70%**	**14**	1.1%*	**247**	**19.80%**	**345**	**27.60%**
**Problem is a sign of personal weakness**	**149**	**11.80%**	**48**	**3.90%**	**86**	6.9%*	**30**	**8.40%**	**104**	2.4%**	**80**	**6.40%**	**54**	4.3%*	**17**	**8.40%**	**77**	**6.20%**	**35**	**2.80%**	**5**	**0.40%**	**62**	**5.00%**	**72**	**5.85**
**Problem is not a real medical illness**	**184**	**14.60%**	**55**	**4.40%**	**111**	8.9%*	**25**	**2.00%**	**141**	11.4%**	**101**	**8.10%**	**65**	5.2%*	**22**	**1.80%**	**107**	**8.60%**	**34**	**2.70%**	**3**	0.2%*	**72**	**7.60%**	**94**	**5.80%**
**People with this problem are dangerous**	**211**	**16.80%**	**57**	**4.60%**	**154**	**12.40%**	**74**	**5.90%**	**137**	**11.00%**	**148**	**11.90%**	**63**	**5.10%**	**19**	**1.50%**	**130**	**10.50%**	**53**	**4.30%**	**9**	**0.70%**	**97**	**9.20%**	**114**	**7.80%**
**Avoid people with this problem**	**245**	**19.50%**	**56**	**4.50%**	**189**	**15.20%**	**65**	**5.20%**	**180**	14.5%**	**179**	**14.40%**	**66**	**5.30%**	**24**	**1.90%**	**139**	**11.20%**	**76**	**6.10%**	**6**	**0.50%**	**120**	**9.70%**	**125**	**10.15**
**People with this problem are unpredictable**	**399**	**31.70%**	**91**	**7.40%**	**308**	25%*	**127**	**10.30%**	**272**	**22.10%**	**287**	**23.30%**	**112**	**9.10%**	**37**	**3.00%**	**241**	**19.50%**	**115**	**9.30%**	**6**	0.5%*	**157**	**12.70%**	**242**	19.6%*
**If I had this problem, I would not tell anyone**	**237**	**18.80%**	**71**	**5.70%**	**166**	**13.40%**	**63**	**5.10%**	**174**	14%**	**171**	**13.80%**	**66**	**5.30%**	**24**	**1.90%**	**142**	**11.40%**	**59**	**4.80%**	**12**	**1.00%**	**98**	**11.20%**	**139**	**7.90%**
**I would not employ someone with this problem**	**87**	**6.90%**	**27**	**2.20%**	**60**	**4.80%**	**24**	**1.90%**	**63**	**5.10%**	**65**	**5.20%**	**22**	**1.80%**	**15**	**1.20%**	**47**	**3.80%**	**19**	**1.50%**	**6**	0.5%*	**42**	**3.40%**	**48**	**3.60%**
**I would not vote for a politician with this problem**	**106**	**8.40%**	**33**	**2.70%**	**73**	**5.90%**	**38**	**3.10%**	**68**	5.5%**	**75**	**6.00%**	**31**	**2.50%**	**9**	**0.70%**	**58**	**4.70%**	**32**	**2.60%**	**7**	**0.60%**	**53**	**4.30%**	**53**	**4.30%**
**DPSS total score (mean ± SD)**	**1.74**	**1.4**	**1.6**	**1.4**	**1.7**	**1.4**	**1.4**	**1.2**	**1.8**	**1.5**	**1.7**	**1.4**	**1.7**	**1.4**	**1.9**	**1.5**	**1.7**	**1.4**	**1.6**	**1.3**	**1.6**	**1.7**	**1.7**	**1.4**	**1.7**	**1.4**
**Statement about personal belief**	**Total (N = 935)**		**Gender**				**Major section**				**Region**				**Economic level**								**Occupation status**			
	**n**		**Male (n = 327)**		**Female (n = 932)**		**Medical (n = 438)**		**Non-Medical (n = 821)**		**City (n = 890)**		**Rural region (n = 369)**		**Low (n = 105)**		**Moderate (n = 748)**		**Good (n = 363)**		**High (n = 43)**		**Worker (n = 542)**		**Non-Worker (n = 717)**	
		**%**	**n**	**%**	**n**	**%**	**n**	**%**	**n**	**%**	**n**	**%**	**n**	**%**	**n**	**%**	**n**	**%**	**n**	**%**	**n**	**%**	**n**	**%**	**n**	**%**
**The person could snap out of the problem**	**602**	**47.80%**	**115**	**9.20%**	**477**	38.2%**	**179**	**14.30%**	**413**	33.1%**	**421**	**33.70%**	**171**	**13.70%**	**37**	**3.00%**	**370**	**29.60%**	**171**	**13.70%**	**14**	1.1%*	**247**	**19.80%**	**345**	**27.60%**
**Problem is a sign of personal weakness**	**149**	**11.80%**	**48**	**3.90%**	**86**	6.9%*	**30**	**8.40%**	**104**	2.4%**	**80**	**6.40%**	**54**	4.3%*	**17**	**8.40%**	**77**	**6.20%**	**35**	**2.80%**	**5**	**0.40%**	**62**	**5.00%**	**72**	**5.85**
**Problem is not a real medical illness**	**184**	**14.60%**	**55**	**4.40%**	**111**	8.9%*	**25**	**2.00%**	**141**	11.4%**	**101**	**8.10%**	**65**	5.2%*	**22**	**1.80%**	**107**	**8.60%**	**34**	**2.70%**	**3**	0.2%*	**72**	**7.60%**	**94**	**5.80%**
**People with this problem are dangerous**	**211**	**16.80%**	**57**	**4.60%**	**154**	**12.40%**	**74**	**5.90%**	**137**	**11.00%**	**148**	**11.90%**	**63**	**5.10%**	**19**	**1.50%**	**130**	**10.50%**	**53**	**4.30%**	**9**	**0.70%**	**97**	**9.20%**	**114**	**7.80%**
**Avoid people with this problem**	**245**	**19.50%**	**56**	**4.50%**	**189**	**15.20%**	**65**	**5.20%**	**180**	14.5%**	**179**	**14.40%**	**66**	**5.30%**	**24**	**1.90%**	**139**	**11.20%**	**76**	**6.10%**	**6**	**0.50%**	**120**	**9.70%**	**125**	**10.15**
**People with this problem are unpredictable**	**399**	**31.70%**	**91**	**7.40%**	**308**	25%*	**127**	**10.30%**	**272**	**22.10%**	**287**	**23.30%**	**112**	**9.10%**	**37**	**3.00%**	**241**	**19.50%**	**115**	**9.30%**	**6**	0.5%*	**157**	**12.70%**	**242**	19.6%*
**If I had this problem, I would not tell anyone**	**237**	**18.80%**	**71**	**5.70%**	**166**	**13.40%**	**63**	**5.10%**	**174**	14%**	**171**	**13.80%**	**66**	**5.30%**	**24**	**1.90%**	**142**	**11.40%**	**59**	**4.80%**	**12**	**1.00%**	**98**	**11.20%**	**139**	**7.90%**
**I would not employ someone with this problem**	**87**	**6.90%**	**27**	**2.20%**	**60**	**4.80%**	**24**	**1.90%**	**63**	**5.10%**	**65**	**5.20%**	**22**	**1.80%**	**15**	**1.20%**	**47**	**3.80%**	**19**	**1.50%**	**6**	0.5%*	**42**	**3.40%**	**48**	**3.60%**
**I would not vote for a politician with this problem**	**106**	**8.40%**	**33**	**2.70%**	**73**	**5.90%**	**38**	**3.10%**	**68**	5.5%**	**75**	**6.00%**	**31**	**2.50%**	**9**	**0.70%**	**58**	**4.70%**	**32**	**2.60%**	**7**	**0.60%**	**53**	**4.30%**	**53**	**4.30%**
**DPSS total score (mean ± SD)**	**1.74**	**1.4**	**1.6**	**1.4**	**1.7**	**1.4**	**1.4**	**1.2**	**1.8**	**1.5**	**1.7**	**1.4**	**1.7**	**1.4**	**1.9**	**1.5**	**1.7**	**1.4**	**1.6**	**1.3**	**1.6**	**1.7**	**1.7**	**1.4**	**1.7**	**1.4**

### Perceived stigma

Participants’ agreements about the items reflecting other people’s beliefs are shown in Table 3. Half of the respondents were most likely to agree that most other people would think that people with depression can snap out of it if they wanted to. When comparing perceived stigma (Table 3) to personal stigma questionnaires (Table 2), About 37.80% of respondents thought that other people consider depression a sign of weakness. In contrast, only 11.80% believed so when expressing their personal belief in this regard. A quarter of non-medical respondents thought most people would not treat depression as a medical illness, and 17.7% believed that most people would consider depressed people dangerous. 38.60% of respondents estimated that most people would find it best for themselves to avoid depressed people in order not to be depressed. In addition, 31.90% thought that most people wouldn’t employ or elect a person with depression. According to Table 2, a far less portion of people will tend to act that way.

**Table 3:** Percentage of participants who “agree” or “strongly agree” with about perceived stigma towards depression patient scale statements.

Statement about Perceived belief	Total (N = 935)		Gender				Major section				Region				Economic level								Occupation status			
	n		Male (n = 327)		Female (n = 932)		Medical (n = 438)		Non-Medical (n = 821)		City (n = 890)		Rural region (n = 369)		Low (n = 105)		Moderate (n = 748)		Good (n = 363)		High (n = 43)		Worker (n = 542)		Non-worker (n = 717)	
		%	n	% (95% CI)	n	% (95% CI)	n	% (95% CI)	n	% (95% CI)	n	% (95% CI)	n	% (95% CI)	n	% (95% CI)	n	% (95% CI)	n	% (95% CI)	n	% (95% CI)	n	% (95% CI)	N	% (95% CI)
**Most people belive that people with depression could snap out of it if they wanted**	**632**	**50.20%**	**132**	**11.30%**	**500**	42.9%**	**235**	**20.20%**	**397**	**34.00%**	**452**	**38.80%**	**180**	**15.40%**	**51**	**4.40%**	**376**	**32.20%**	**187**	**16.00%**	**18**	**1.50%**	**273**	**23.40%**	**359**	**30.80%**
**Most people believe that Depression is a sign of personal weakness.**	**476**	**37.80%**	**107**	**9.40%**	**369**	32.3%*	**228**	**20.00%**	**248**	21.7%**	**356**	**31.20%**	**120**	10.5%*	**36**	**3.20%**	**295**	**25.90%**	**128**	**11.20%**	**17**	**1.50%**	**207**	**18.10%**	**269**	**23.60%**
**Most people believe that Depression is not a medical illness.**	**518**	**41.10%**	**118**	**10.50%**	**400**	35.5%*	**231**	**20.50%**	**287**	25.5%**	**375**	**33.30%**	**143**	**12.70%**	**41**	**3.60%**	**313**	**27.80%**	**146**	**13.00%**	**18**	**1.60%**	**229**	**20.30%**	**289**	**25.60%**
**Most people believe that people with Depression are dangerous.**	**353**	**28.00%**	**74**	**6.60%**	**279**	24.8%**	**154**	**13.70%**	**199**	17.7%**	**257**	**22.90%**	**96**	**8.50%**	**27**	**2.40%**	**211**	**18.80%**	**101**	**9.00%**	**14**	**1.20%**	**150**	**13.40%**	**203**	**18.10%**
**Most people believe that it is best to avoid people with Depression so that you don’t become depressed yourself.**	**486**	**38.60%**	**99**	**8.80%**	**387**	34.5%**	**213**	**19.00%**	**273**	24.3%**	**364**	**32.40%**	**122**	10.9%*	**34**	**3.00%**	**284**	**25.30%**	**155**	**13.80%**	**13**	**1.20%**	**209**	**18.60%**	**277**	**24.70%**
**Most people believe that people with Depression are unpredictable.**	**361**	**28.70%**	**84**	**7.50%**	**277**	24.9%*	**124**	**11.10%**	**237**	21.3%**	**254**	**22.80%**	**107**	**9.60%**	**29**	**2.60%**	**221**	**19.80%**	**98**	**8.80%**	**13**	**1.20%**	**162**	**14.50%**	**199**	**17.90%**
**If they had Depression most people would not tell anyone.**	**513**	**40.70%**	**113**	**10.10%**	**400**	35.8%**	**216**	**19.30%**	**297**	26.6%**	**371**	**33.20%**	**142**	**12.75**	**37**	**3.30%**	**306**	**27.40%**	**153**	**13.70%**	**17**	**1.50%**	**220**	**19.70%**	**293**	**26.20%**
**Most people would not employ someone they knew had been affected with Depression**	**402**	**31.90%**	**84**	**7.50%**	**318**	28.4%**	**186**	**16.60%**	**216**	19.3%**	**300**	**26.80%**	**102**	9.1%*	**30**	**2.70%**	**234**	**20.90%**	**122**	**10.90%**	**16**	**1.40%**	**179**	**16.00%**	**223**	**19.90%**
**Most people would not vote for a politician they knew had been affected with Depression**	**401**	**31.90%**	**79**	**7.10%**	**322**	28.8%**	**178**	**15.90%**	**223**	19.9%**	**289**	**25.80%**	**112**	**10.00%**	**33**	**3.00%**	**235**	**21.00%**	**118**	**10.60%**	**15**	**1.30%**	**178**	**15.90%**	**223**	**19.90%**
**DPSS total score (mean ± SD)**	**3.7**	**2.90**	**2.9**	**2.70%**	**3.9**	2.9%**	**4.2**	**3**	**3.3**	2.7**	**3.8**	**2.9**	**3.4**	**2.8**	**3.4**	**2.9**	**3.7**	**2.8**	**3.6**	**2.9**	**3.4**	**3.4**	**3.6**	**2.8**	**3.7**	**2.8**

### Social distance

Participants’ endorsements for "probably unwilling" or "definitely unwilling" to have contact with the depressed person are shown in Table 4. In general, more than half of respondents won’t live next door to depressed people if they had to choose, more than half won’t work close to them, 36.90% won’t make friends with them. More than 90% won’t marry one of them.

**Table 4:** Percentage of participants who “probably unwilling” or “definitely unwilling” to have contact with depression patient.

Statement about personal belief (SDS)	Total (N = 1259)		Gender				Major section				Region				Economic level								Occupation status			
	N		Male (n = 327)		Female (n = 932)		Medical (n = 438)		Non-Medical (n = 821)		City (n = 800)		Rural region (n = 369)		Low (n = 105)		Moderate (n = 748)		Good (n = 363)		High (n = 43)		Worker (n = 542)		Non-Worker (n = 717)	
		%	n	%	n	%	n	%	n	%	n	%	n	%	n	%	n	%	n	%	n	%	n	%	n	%
**Live next door**	**669**	**53.10%**	**179**	**14.00%**	**496**	**40.00%**	**250**	**20.20%**	**419**	33.8%*	**477**	**32.20%**	**171**	**15.50%**	**55**	**4.40%**	**378**	**30.50%**	**208**	**16.80%**	**28**	2.3%*	**401**	**24.40%**	**268**	21.6%**
**Spend the evening socializing**	**487**	**38.70%**	**135**	**10.90%**	**352**	**28.40%**	**204**	**16.50%**	**283**	22.9%**	**345**	**27.90%**	**142**	**11.50%**	**40**	**3.20%**	**282**	**22.80%**	**145**	**11.70%**	**20**	**1.60%**	**274**	**22.10%**	**213**	**17.20%**
**Make friends**	**465**	**36.90%**	**133**	**10.80%**	**332**	**26.90%**	**172**	**13.90%**	**293**	**23.80%**	**328**	**26.60%**	**137**	**11.10%**	**43**	**3.50%**	**278**	**22.50%**	**126**	**10.20%**	**18**	**1.50%**	**270**	**21.90%**	**195**	**15.80%**
**Work closely**	**675**	**53.60%**	**176**	**14.30%**	**499**	**40.40%**	**228**	**18.50%**	**447**	**36.20%**	**474**	**38.40%**	**201**	**16.30%**	**55**	**4.50%**	**391**	**31.70%**	**201**	**16.30%**	**28**	**2.30%**	**413**	**33.50%**	**262**	21.2%**
**Marry into family**	**1134**	**90.10%**	**304**	**24.70%**	**830**	**67.30%**	**398**	**32.30%**	**736**	**59.70%**	**808**	**65.50%**	**326**	**26.40%**	**96**	**7.80%**	**669**	**54.30%**	**332**	**26.90%**	**37**	**3.00%**	**645**	**52.30%**	**489**	**39.70%**
**DSS total score (mean ± SD)**	**2.7**	**1.30%**	**2.8**	**1.30%**	**2.7**	**1.30%**	**2.8**	**1.40%**	**2.7**	1.3%*	**2.7**	**1.30%**	**2.7**	**1.30%**	**2.7**	**1.30%**	**2.7**	**1.30%**	**2.8**	**1.30%**	**3**	**1.40%**	**2.6**	**1.30%**	**2.8**	**1.40%**

### Predictors for stigma and social distance by multiple linear regression analysis

We performed multiple logistic regression to analyze the predicted relationship between the three scales and other demographic variables. We found that almost variables couldn’t be linked in a statistically significant expected test for all scales, except the significant section (Medical student or non-medical student) on three scales, sex variable in depression perceived stigma scale, and the current region or location according to social distance scale. The adjusted R square values for DPSS, DPSS*, and SDS were 0.19, 0.050, and 0.005, respectively (Table 5).

**Table 5:** Predictors for stigma and social distance by multiple linear regression analysis.

Dependent variable	Predictors	B	t	P*	R	R^2^	Adj.R^2^
**DPSS**					0.165	0.24	0.19
** Gender**		0.02	0.2	0.83			
** Age**		0.01	0.9	0.33			
** Major section**		0.44	4.9	0.00			
** Economic level**		-0.06	-1.06	**0.28**			
** Region settings**		-0.02	-0.23	0.81			
** Occupation status**		-0.056	-0.6	0.54			
DPSS*					0.23	0.056	0.050
** Gender**		1.12	5.6	0.000			
** Age**		0.01	0.85	0.3			
** Major section**		-1.06	-5.7	0.000			
** Economic level**		-0.17	-1.3	0.18			
** Region settings**		0.14	0.78	0.43			
** Occupation status**		-0.15	-0.76	0.44			
**SDS**					0.09	0.01	0.005
** Gender**		-0.11	-1.27	0.2			
** Age**		-0.005	-0.43	0.6			
** Major section**		-0.14	-1.6	0.09			
** Economic level**		0.05	0.94	0.3			
** Region settings**		-0.17	-2.1	0.03			
** Occupation status**		0.28	0.32	0.7			

### Participants’ usual sources of mental health knowledge

**Fig 1:**
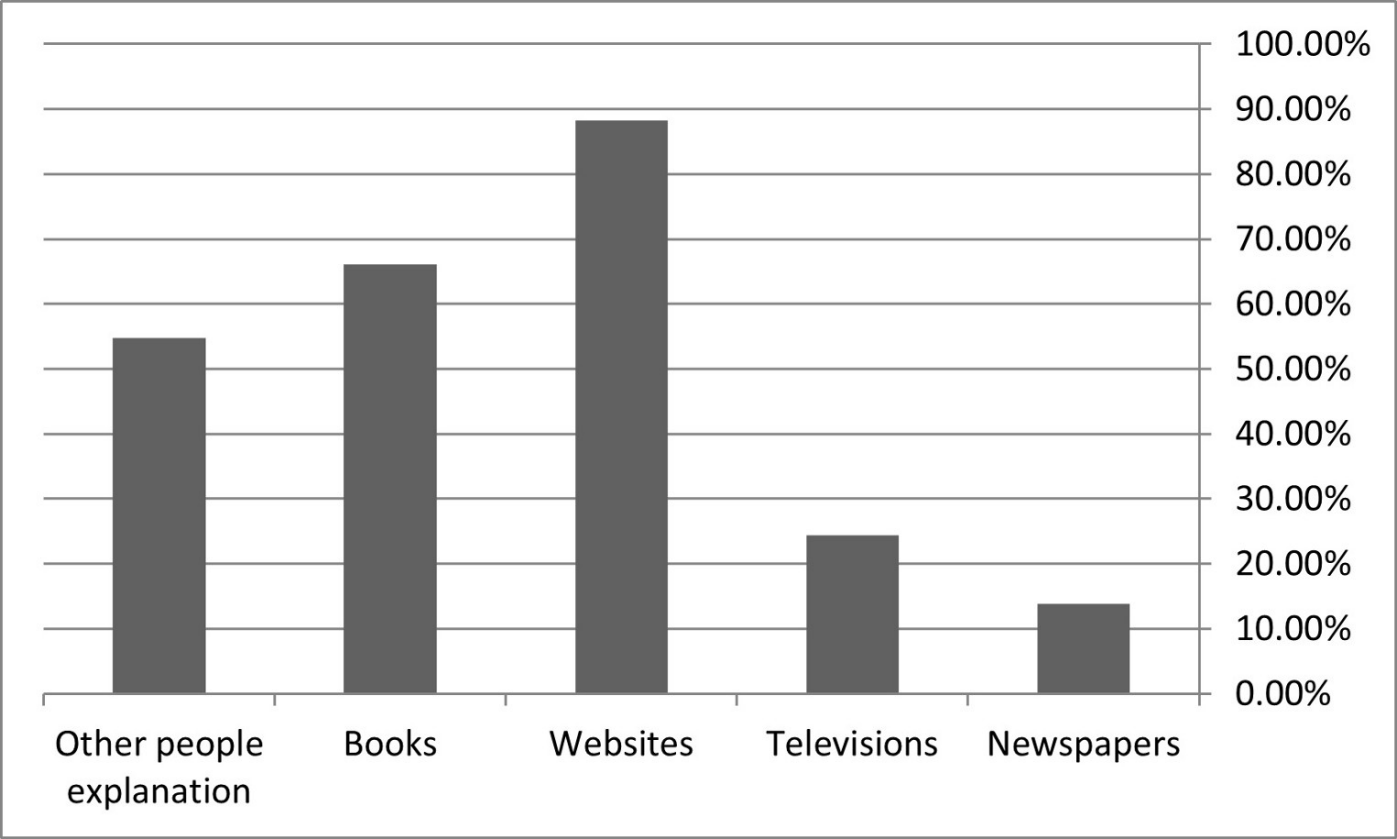
Participants’ usual sources of mental health knowledge.

Showing in Fig 1, the most dependent source of information towards mental health knowledge was internet websites (85.80%). Books (64.40%), people explanation (53.10%), television (23.90%), and newspapers (11.30%) were also reliable sources of information, respectively.

In Table 6, we asked the respondents about people, medications, and other interventions that they might find helpful for depressed people. Regarding most beneficial people, 79.3% agreed that a psychiatrist would greatly assist such mental health issues. About 59.8% thought that help from a close family member would be beneficial, and 59.3% consented to pray to God as a helpful solution.

**Table 6:** Recommended helpful interventions by the participants.

**People who can help**		
**A typical GP or family doctor**	575	45.9%
**A pharmacist**	140	11.2%
**A counsellor**	349	27.9%
**A social worker**	335	26.7%
**A telephone counselling service**	126	10.1%
**A psychiatrist**	994	79.3%
**A psychiatric nurse**	418	33.4%
**A clinical psychologist**	704	56.2%
**Help from close family**	749	59.8%
**Help from close friends**	849	67.8%
**An herbalist**	42	3.4%
**Pray to god for help**	743	59.3%
**Medication which can help**		
**Vitamins and mineral**	562	47.5%
**Laxatives such as lactulose or Senna**	22	1.9%
**Tonics or herbal medicines**	157	13.3%
**Antibiotics**	86	7.3%
**Antidepressants**	897	75.8%
**Pain relievers such as aspirin or acetaminophen**	112	9.5%
**Sleeping pills**	188	15.9%
**Antipsychotics**	172	14.5%
**Tranquillizer such as diazepam**	331	28%
**Anxiolytics**	662	55.9%
**Other Inventions**		
**Becoming physically more active, such as playing more sports, or doing a lot more walking or gardening.**	1013	82%
**Undergoing electro-convulsive therapy.**	690	55.9%
**Getting out more.**	819	66.3%
**Staying at home and resting.**	213	17.2%
**Having an occasional alcoholic drink to relax.**	68	5.5%
**Psychotherapy**	794	64.3%
**Attending courses or relaxation, stress management, meditation, or yoga**	552	44.7%
**Cutting out alcohol altogether.**	68	5.5%
**Massage to relax.**	352	28.5%
**Acupuncture therapy.**	71	5.7%
**Being admitted to a psychiatric hospital.**	103	8.3%
**Reading about people with similar problems and how they have dealt with them.**	690	55.9%
**Going on a special diet or avoiding certain foods.**	352	28.5%
**Aromatic therapy.**	94	7.6%
**Hypnosis**	103	8.3%
**Being admitted to a psychiatric ward or general hospital.**	117	9.5%
**Help methods**		
**Encourage the person to seek help.**	705	59.1%
**Accompany the person to professional help.**	666	54.4%
**Contact professional help on the person`s behalf.**	133	10.9%
**Listen with the person**	723	59.1%
**Encourage the person to see a community physician.**	313	25.6%
**Encourage the person to see a counsellor.**	319	26.1%
**Encourage the person to see psychiatrist.**	768	62.7%
**Give advice.**	629	51.4%
**Encourage the person to go to hospital.**	126	10.3%
**Encourage the person to see psychologist.**	651	53.2%
**Encourage the person to go to a mental health clinic.**	164	13.4%
**Ask if the person wants help**	615	50.2%
**Assess the problem/risk of harm.**	310	25.3%
**Do an intervention.**	123	10%
**Cheer the person up/boost the person`s confidence.**	755	61.7%
**Tell the person`s parents or family.**	325	26.6%
**Seek information for the person.**	547	46.9%
**Help the person make new friends.**	599	48.9%
**Help with chores/work.**		
**Provide general support (e.g. practical emotional).**	638	52.1%
**Spend time/socialize with the person.**	681	55.6%
**Encourage the person to become physically active.**	699	57.1%

Antidepressants (75.8%), anxiolytics (55.9%), and vitamins (47.5%) were the top three medications that respondents chose and thought to be most helpful, respectively.

Out of all the activities that have been suggested to aid depression, encouraging physical activity was chosen to be the most helpful intervention (82%). On the other hand, most respondents didn’t think cutting out alcohol would benefit much (5.5%).

**Fig 2:**
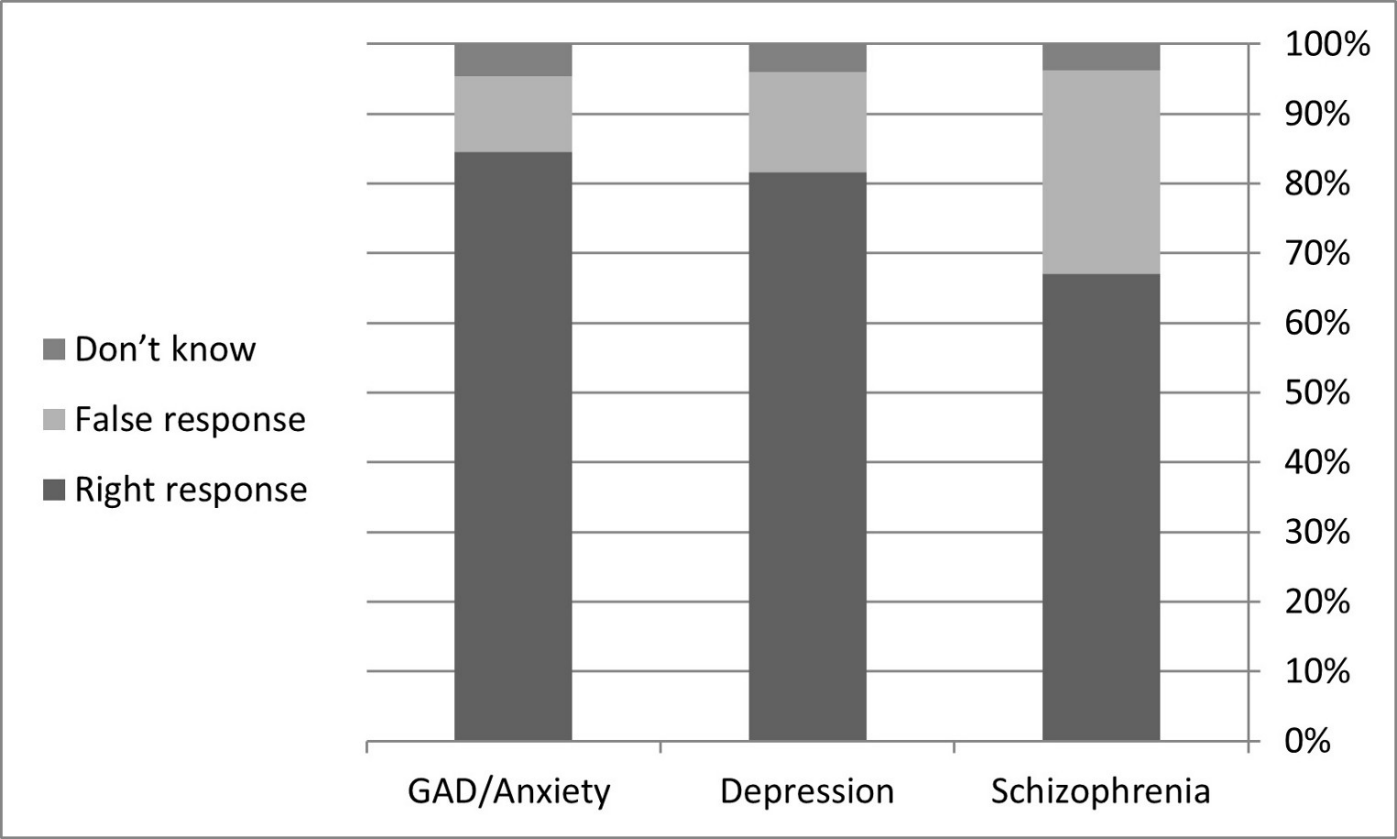
Supporting information: (Knowledge towards the three mental disorders) [depression].

Finally, we reported good percentages of the correct knowledge responses toward diagnosing three selected psychiatric disorders. 14.30%, 15.20%, and 27.90% were the percentages of the incorrect answers in awareness of diagnosis Anxiety, Depression, and Schizophrenia, respectively (Fig 2).

## Discussion

To our knowledge, this is the first study to use a case vignette to investigate gender (male and female), major (medical and non-medical), region (rural and city), occupation (worker and non-worker), and economic status (low, moderate, good, high) differences in Syrian students’ stigma attitudes toward people with depression and the desire for social distancing. In each result, we attempted to investigated enough similar papers with close questionnaires, and discussed the similarities and the differences. However, we did not discuss studies that involved Syrians in asylum countries [32, 33].

The study found a higher level of stigma and desire for social distance among Syrian students toward depressive people than in other studies like muzzling et al. [40], where Italian people who experienced depression either directly or indirectly hold less stigmatizing attitudes. The percentage of the respondents who agreed that “depressive people are dangerous” was higher in China (60%) [41], Qatar (60%) [42], brazil (56%) [43], Italy (27%) [37] and united states (33%) [44] than in the present study (18.8%) and this may be because the onset of depression is due mainly to stressful life conditions other than hereditary causes [40]. A low percentage of the respondents (18.8%) stated that “if I have this problem, I will not tell anyone,” compared to a widespread belief among the Italian population (75%) who tend to hold their condition in solitude [40] and this in line with Australian people who have the belief of helpfulness of self-reliance [45]. Compared to the non-medical students (2.4%), medical students (8.4%) agreed more that “depression is a sign of personal weakness,” and this is in line with He et al. [41], where medical students (50%) agreed more about that subscale than non-medical (38%). This may be due to the that psychiatric education focuses mainly on professional knowledge for diagnosis and treatment but neglects the humanistic and emotional concerns that reduce the stigma towards the mentally ill patients [41] and may be associated with their belief that depressive patients are unpredictable, dangerous, and find it hard to control themselves [46].

To be more specific, respondents disagreed that “live next door to depressive neighbor” (50%), “spending the event socializing with depressive ones” (38.7%), “make depressive people close friends” (36.9%), “marry into a family with a history of depression” (90%) and “work closely with mentally ill people” (53.6%). This are line in with other studies like He et al. [41], where (71.1%) of the respondents agreed that they would not marry someone who is depressed, and (45.1%) of them would not work closely with them. The results are line in with other studies like He et al. [41] and Anosike et al. [47]. For example, a study in Nigeria found that 49% of the undergraduate students agreed that “I would be against any daughter of mine marrying a man who had been to the hospital to see a psychiatrist about mental problems.”The cause of keeping social distance may be due to the belief That depressive people are dangerous [41]. A line of research studies that also suggest that people with depression (or other mental illnesses) are difficult seems to generate increased social distancing [48, 49].

Most of the respondents (79.5%) hold the belief that mentally-ill people would gain great assistance from the psychiatrists, which is in line with other studies such as Holzinger et al. [50] and muzzling et al. [40], which reported that in Italy most of the respondents hold the attitude of seeking professional help as a first choice and this is compared to Ibrahim et al. [51] which reported that most of the students with mental health problems seek support first from non-medical personal such as peers and family. This indicates that social support represents a cornerstone step in achieving professional support in the future. Avoiding seeking professional help may be due to considering that as a threat to self-esteem, a sign of weakness, acceptance of failure, and the fear of being labeled as a mentally ill patient [51–53]. In addition to the previous causes, Özmen et al. [54] reported that respondents agreed that psychological counseling and social support for mentally ill patients are more effective than treatment by medications considered harmful and addictive.

In this study female students hold higher level of stigmatization and social distance atitude in all subscales than the male students compared to Anderson et al. [48] which reported that male students were more unwilling to work closely with depressive people, Korszun et al. [55] reported that female students hold a greater sympathy towards mentally ill patients than male students and He et al. which reported that male students holds higher stigamtizating and social distance attitude than female students in all subscales except “work closely” and “marry” with depressive person subscales (40.3% vs 47.5% and 68.5% vs 72.5% respectively). This may be attributed to the already imbalanced gender ratio in Syrian schools and that female students and the fact that females hold better knowledge about mental illness and subsequentaly more kind towards mentally ill patients [55].

Oliveira et al. [56] reported that the more the contact with mentally ill people, the lower the stigma. Unlike other physicians, psychiatrists and physicians with a relative of mental illness show lower stigma and higher scores on pity and help. This is explained by contact hypothesis and the fact that other specialities may contact the mentally ill patient in more serious conditions in the emergency room in virtue of their mental illness. As regard the students, the study reported that the more the education in psychiatry, the less the stigma. The level of the stigma has decreased significantly between the students after taking the psychiatry rotation which managed to change their thoughts and beliefs about the mantally ill patients.The study limitations include small sample size, the cross sectional design of the study doesn’t allow the follow up to observe the change in beliefs towards the mentally ill patient overtime and the old version of the AQ-27 in Portuguese.

He et al. [41] investigated the gender and tha major differences among college students in tha attitude towards depressive people. The study stated that unlike female students, male students agreed more that if they had depression, they wouldn’t tell anyone.With female predominence, Most of the respondents would be unwilling to marry or work with depressive peoples. The respondents thought that depressive people are ineloquent, unpredictable, danger and couldn’t control their behavoir. Thus they hold social distance attitude towards mentally ill patients. The study also reported that psychiatric education focused mainly on professional knowledge acquisition like how to diagnose and treat mentally ill patients but neglect the correction of the negative attitudes towards the patients and lack the humanistic and emotional concerns may contribute to the stigma.

Wu et al. [57] investigated the attitude towards depressive patients among the non-mental health professionals and reported that they are unwilling to hire a relative or marry someone with mental health problems like schizophrenia, depression or GAD (generalized anxiety disorder). They believe that they are dangerous, unpredicable and may hurt themselfies and others.The participants admitted that they gained such negative attitude through the socail media mainly from newspapers.

Palou et al. [58] reported that over the training course of the undergraduate nursing students in the mental health field, their attitudes towards mentally ill patients were significantly improved. It was also found that psychiatric education including the issues of stigmatization is associated with better knowledge acquisition and positive attitudes towards the people with mental health problems. The study has some limitations such as it included only the students so the results cannot be generalizable and not all socioeconomic characteristics of the participants were considered.

Dey et al. [59] found that males are more stigmatizing than females towards their peers with mental health problem. This may be due to the fact that males consider the mental health problem a sign of weakness not sickness and the fact that males should be able to manage their psychiatric problems on their owns. For the “unpredictible and dangerous” factor, authors stated that the cause of such attitude is likely due to the stereotypical image of masculinity. Males with mental health problems are more stigmatized than females. This may be due to the methodological design of the used survey that makes the character’s gender of the vignette was similar to the gender of the participants.

### The limitations

The stigmatizing attitudes were investigated in the view of hypothetical scenarios, not towards real-life people, and no causal conclusion could be withdrawn as the study design is cross-sectional, not all social characteristics of the Syrian population were considered, and the people of the study are only students so the results cannot be generalized. In addition, Depression Stigma Scales and Social distance scale have not been validated in Arab populations.

## Conclusion

A significant proportion of the Syrian students have a high level of stigma attitudes towards depressed persons. But massive numbers of Syrian students are willing to deal with these persons in their lives. Multiple recommendations should be made to improve the public health attitudes towards these patients, incredibly depressed persons. Campaigns are required to invest more efforts into wider areas of the country; this includes schools, universities, health centers, and hospitals. Now, It is more important than ever to start looking for more appropriate methods to implement anti-stigma ideologies into the community. It is also clear now that planning on increasing the psychological health workers capacity is vital for overall mental health issues in Syria. One of the most causes may be the deteriorated situation in Syria after the humanity war for 11 years. The leaders of the global humanitarian organizations should support the mental health psychiatry in Syria through awareness programs for suitable dealing with mental health patients, improving the current infrastructures of the psychiatric hospitals, and supporting mental centers.

## Supporting information

S1 Table The English version of the used questionnaire. (DOCX)

S1 File (ZIP)
