# Correction: Stigmatizing attitudes towards depression among university students in Syria

**DOI:** 10.1371/journal.pone.0293795

**Published:** 2023-10-26

**Authors:** Sarya Swed, Sheikh Sohib, Noheir Ashraf Ibrahem Fathy Hassan, Mohammad Badr Almoshantaf, Sidra Mhd Sammer Alkadi, Yossef Hassan AbdelQadir, Nancy Ibrahim, Lina Taha Khair, Agyad Bakkour, Ali Hadi Hussein Muwaili, Dhuha Hadi Hussein Muwaili, Fatima Abubaker Abdalla Abdelmajid, Eman Mohammed Sharif Ahmad, Muhammad Mainuddin Patwary, Bisher Sawaf, Mhd Kutaiba Albuni, Elias Battikh, Nashaat Kamal Hamdy

The second author’s name is spelled incorrectly. The correct name is: Sheikh Shoib. The correct citation is: Swed S, Shoib S, Fathy Hassan NAI, Almoshantaf MB, Alkadi SMS, AbdelQadir YH, et al. (2022) Stigmatizing attitudes towards depression among university students in Syria. PloS ONE, 17(9), e0273483. https://doi.org/10.1371/journal.pone.0273483

The data collection group is missing from the Acknowledgments section. The Acknowledgments should list the following individuals from the data collection group: Haidara Bohsas (Faculty of Medicine, Aleppo University, Aleppo, Syria; haidara.bohsas@gmail.com) Hidar Alibrahim (Faculty of Medicine, Aleppo University, Aleppo, Syria; haideralibrahem1999@gmail.com) Shahm alsakka (Faculty of Medicine, Hama University, Hama, Syria; shahemalsakka@gmai.com) Mhd Noor Tahawi (Faculty of Medicine, Albaath University; noor251998@gmail.com) Mulham Al-Zahran (Faculty of Medicin, Hama University, Syria; mulhamz2000@gmail.com) Joudi Anadani (Faculty of Medicine, Aleppo University, Syri; joudmglj99@gmail.com) Nader Ebrahim (Faculty of Medicine, Hama Univesity, Hama, Syria; nadern567@gmail.com)
