# Supplementary material for: Stigmatizing attitudes towards depression among university students in Syria
Source: PLoS One. 2022 Sep 15;17(9):e0273483. doi: 10.1371/journal.pone.0273483 (PMC9477282; doi:10.1371/journal.pone.0273483)
Supplement: S1 Table — (DOCX) [file pone.0273483.s001.docx]

**S1 Table 1 : The Questionnaire**

| Demographic baseline characteristics | | | | | | | | | | | | |
| --- | --- | --- | --- | --- | --- | --- | --- | --- | --- | --- | --- | --- |
| Age |  | | | | | | | | | | | |
| Gender | - Male | | | | | | - Female | | | | | |
| Social status | - Single | | | - Married | | | - Divorced | | | - Widower | | |
| Education | - Medical student | | | | | | - Non-medical student | | | | | |
| Economic level | - bad | | | - middle | | | - good | | | - high | | |
| The university stage | - **1** | - **2** | | | - **3** | | - **4** | | - **5** | | | - **6** |
| Region | - City | | | | | | - Rural | | | | | |
| Occupation level | - Yes | | | | | | - No | | | | | |
| Live with | - family | | - With father | | | - With mother | | - With friend | | | - alone | |
| Immigrant status | - Yes | | | | | | - No | | | | | |
| History of depression | - Yes | | | | | | - No | | | | | |
| Current psychological treatment | - Yes | | | | | | - No | | | | | |
| Current pharmacological treatment | - Yes | | | | | | - No | | | | | |
| Personal Stigma towards mental health patient scale. | | | | | | | | | | | | |
| People with depression could snap out of it if they wanted. | - Strongly disagree | | - Disagree | | | - Neutral | | - Agree | | | - Strongly agree | |
| Depression is a sign of personal weakness. | - Strongly disagree | | - Disagree | | | - Neutral | | - Agree | | | - Strongly agree | |
| Depression is not a real medical illness. | - Strongly disagree | | - Disagree | | | - Neutral | | - Agree | | | - Strongly agree | |
| People with depression are dangerous. | - Strongly disagree | | - Disagree | | | - Neutral | | - Agree | | | - Strongly agree | |
| It is best to avoid people with depression so you don’t become depressed yourself. | - Strongly disagree | | - Disagree | | | - Neutral | | - Agree | | | - Strongly agree | |
| People with depression are unpredictable. | - Strongly disagree | | - Disagree | | | - Neutral | | - Agree | | | - Strongly agree | |
| If I had depression, I would not tell anyone. | - Strongly disagree | | - Disagree | | | - Neutral | | - Agree | | | - Strongly agree | |
| I would not employ someone if I knew they had been depressed. | - Strongly disagree | | - Disagree | | | - Neutral | | - Agree | | | - Strongly agree | |
| I would not vote for a politician if I knew they had been depressed. | - Strongly disagree | | - Disagree | | | - Neutral | | - Agree | | | - Strongly agree | |

| Perceived Stigma towards mental health patient scale. | | | | | |
| --- | --- | --- | --- | --- | --- |
| Most people believe that people with depression could snap out of it if they wanted. | - Strongly disagree | - Disagree | - Neutral | - Agree | - Strongly agree |
| Most people believe that depression is a sign of personal weakness. | - Strongly disagree | - Disagree | - Neutral | - Agree | - Strongly agree |
| Most people believe that depression is not a medical illness. | - Strongly disagree | - Disagree | - Neutral | - Agree | - Strongly agree |
| Most people believe that people with depression are dangerous. | - Strongly disagree | - Disagree | - Neutral | - Agree | - Strongly agree |
| Most people believe that it is best to avoid people with depression so that you don’t become depressed yourself. | - Strongly disagree | - Disagree | - Neutral | - Agree | - Strongly agree |
| Most people believe that people with depression are unpredictable. | - Strongly disagree | - Disagree | - Neutral | - Agree | - Strongly agree |
| If they had depression, most people would not tell anyone. | - Strongly disagree | - Disagree | - Neutral | - Agree | - Strongly agree |
| Most people would not employ someone they knew had been depressed. | - Strongly disagree | - Disagree | - Neutral | - Agree | - Strongly agree |
| Most people would not vote for a politician they knew had been depressed. | - Strongly disagree | - Disagree | - Neutral | - Agree | - Strongly agree |

|  |  | Social Distance | | | |
| --- | --- | --- | --- | --- | --- |
| Live next door | unwillingness | | willingness | strongly willingness | strongly unwillingness |
| Spend the evening socializing | unwillingness | | willingness | strongly willingness | strongly unwillingness |
| Make friends | unwillingness | | willingness | strongly willingness | strongly unwillingness |
| Work closely | unwillingness | | willingness | strongly willingness | strongly unwillingness |
| Marry into family | unwillingness | | willingness | strongly willingness | strongly unwillingness |

| Participants’ Usual Sources of Mental Health Knowledge. | | |
| --- | --- | --- |
| How do you usually learn about mental health issues? | | |
| Newspapers | - Yes | - No |
| Televisions | - Yes | - No |
| Websites | - Yes | - No |
| Books | - Yes | - No |
| Other people explanation | - Yes | - No |

| Helpfulness of intervention. | | |
| --- | --- | --- |
| People who can help: (multiple answers) | | |
| - A typical GP or family doctor | - A telephone counselling service | - Help from close family |
| - A pharmacist | - A psychiatrist | - Help from close friends |
| - A counsellor | - A psychiatric nurse | - An herbalist |
| - A social worker | A clinical psychologist | - Pray to god for help |
| Medication which can help: : (multiple answers) | | |
| - Vitamins and mineral | - Antidepressants | - Antipsychotics |
| - Laxatives such as lactulose or Senna | - Pain relievers such as aspirin or acetaminophen | - Tranquillizer such as diazepam |
| - Tonics or herbal medicines | - Sleeping pills | - Anxiolytics |
| - Antibiotics |  |  |
| Other Inventions: : (multiple answers) | | |
| - Becoming physically more active, such as playing more sports, or doing a lot more walking or gardening. | - Attending courses or relaxation, stress management, meditation, or yoga | - Reading about people with similar problems and how they have dealt with them. |
| - Undergoing electro-convulsive therapy. | - Cutting out alcohol altogether. | - Going on a special diet or avoiding certain foods. |
| - Getting out more. | - Massage to relax. | - Aromatic therapy. |
| - Staying at home and resting. | - Acupuncture therapy. | - Hypnosis |
| - Having an occasional alcoholic drink to relax. | - Being admitted to a psychiatric hospital. | - Being admitted to a psychiatric ward or general hospital. |
| - Psychotherapy |  |  |
| Help methods: : (multiple answers) | | |
| - Encourage the person to seek help. | - Encourage the person to go to hospital. | - Tell the person`s parents or family. |
| - Accompany the person to professional help. | - Encourage the person to see psychologist. | - Seek information for the person. |
| - Contact professional help on the person`s behalf. | - Encourage the person to go to a mental health clinic. | - Help the person make new friends. |
| - Listen with the person | - Ask if the person wants help | - Help with chores/work. |
| - Encourage the person to see a community physician. | - Assess the problem/risk of harm. | - Provide general support (e.g. practical emotional). |
| - Encourage the person to see a counsellor. | - Do an intervention. | - Spend time/socialize with the person. |
| - Encourage the person to see psychiatrist. | - Cheer the person up/boost the person`s confidence. | - Encourage the person to become physically active. |
| - Give advice. |  |  |

| Supporting Information: (Knowledge towards the three mental disorders) What is the diagnosis of this person? | | | |
| --- | --- | --- | --- |
| Test number 1: | | | |
| Ahmad is 24 and lives at home with his parents. He has had a few temporary jobs since finishing school but is now unemployed. Over the last six months he has stopped seeing his friends, and has begun locking himself in his bedroom and refusing to eat with the family or to have a bath. His parents also hear him walking about in his bedroom at night while they are in bed. Even though they know he is alone, they have heard him shouting and arguing as if someone else is there. When they try to encourage him to do more things, he whispers that he won’t leave home because he is being spied upon by the neighbours. They realize he is not taking drugs because he never sees anyone or goes anywhere. | | | |
| The Diagnosis is: | - Schizophrenia | - Depression | - Anxiety |
| Test number 2: | | | |
| Lona is 26 years old. She has been feeling unusually sad and miserable for the last few weeks. Even though she is tired all the time, she has trouble sleeping nearly every night. Si Li doesn’t feel like eating and has lost weight. She can’t keep her mind on her work and puts off making any decisions. Even day-to-day tasks seem too much for her. This has come to the attention of Li’s boss who is concerned about her lowered productivity. | | | |
| The Diagnosis is: | - Schizophrenia | - Depression | - Anxiety |
| Test number 3: | | | |
| Lana is 45 years old and she is often worried. She worries a great deal about her job performance, her children’s well-being, and her relationships with men. In addition, she worries about a variety of minor matters such as getting to appointments on time, keeping her house clean, and maintaining regular contact with family and friends. It takes Wu Wang longer than necessary to accomplish tasks because she worries about making decisions. Wu Wang has trouble sleeping at night and finds that she is exhausted during the day and irritable with her family. | | | |
| The Diagnosis is: | - Schizophrenia | - Depression | - Anxiety |
